# Overlapping Protein-Encoding Genes in *Pseudomonas fluorescens* Pf0-1

**DOI:** 10.1371/journal.pgen.1000094

**Published:** 2008-06-13

**Authors:** Mark W. Silby, Stuart B. Levy

**Affiliations:** Center for Adaptation Genetics and Drug Resistance, Department of Molecular Biology and Microbiology, Tufts University School of Medicine, Boston, Massachusetts, United States of America; Baylor College of Medicine, United States of America

## Abstract

The annotated genome sequences of prokaryotes seldom include overlapping genes encoded opposite each other by the same stretch of DNA. However, antisense transcription is becoming recognized as a widespread phenomenon in eukaryotes, and examples have been linked to important biological processes. *Pseudomonas fluorescens* inhabits aquatic and terrestrial environments, and can be regarded as an environmental generalist. The genetic basis for this ecological success is not well understood. In a previous search for soil-induced genes in *P. fluorescens* Pf0-1, ten antisense genes were discovered. These were termed ‘cryptic’ genes, as they had escaped detection by gene-hunting algorithms, and lacked easily recognizable promoters. In this communication, we designate such genes as ‘non-predicted’ or ‘hidden’. Using reverse transcription PCR, we show that at each of six non-predicted gene loci chosen for study, transcription occurs from both ‘sense’ and ‘antisense’ DNA strands. Further, at least one of these hidden antisense genes, *iiv14*, encodes a protein, as does the sense transcript, both identified by poly-histidine tags on the C-terminus of the proteins. Mutational and complementation studies showed that this novel antisense gene was important for efficient colonization of soil, and multiple copies in the wildtype host improved the speed of soil colonization. Introduction of a stop codon early in the gene eliminated complementation, further implicating the protein in colonization of soil. We therefore designate *iiv14* “*cosA*”. These data suggest that, as is the case with eukaryotes, some bacterial genomes are more densely coded than currently recognized.

## Author Summary

Sequenced bacterial genomes provide a vast resource for research fields such as pathogenesis, drug discovery, and microbial ecology. Once sequenced, the genes within a genome are predicted using computational and manual methods. An assumption underlying both approaches is that any given length of DNA encodes only a single gene. This concept has been challenged by findings in eukaryotic genomes, and in bacterial plasmids and viruses where it is known that some stretches of DNA specify both ‘sense’ and ‘antisense’ RNA molecules. In prokaryotic cells there is little information regarding the potential of the genome to code two genes within the same stretch of DNA. We show that in the bacterium *Pseudomonas fluorescens* Pf0-1, both strands of DNA are transcribed at six locations in the genome, and that at one of these locations (*iiv14*), two different proteins are specified by the same piece of DNA. At the *iiv14* locus, we demonstrate that the newly identified gene (antisense to the predicted gene) functions to promote colonization of soil, and name this gene *cosA*. Our findings indicate that bacterial genomes have more genes than currently thought, and important genes that have escaped detection occupy the same stretch of DNA as known genes.

## Introduction

The genetic basis for ecological success is not well understood, yet has practical and fundamental significance. The environmental versatility of *P. fluorescens*, coupled with secondary metabolism that enables strains to antagonize plant pathogenic fungi or degrade organic pollutants, makes this species an important and relevant model for investigating environmental fitness and applications such as biocontrol and bioremediation. Furthermore, insight into complex ecosystem interactions is enhanced by knowledge of fitness determinants of the individual players.

To expand the understanding of genes functioning to promote soil fitness, we examined gene activity by evaluating expression when introduced into soil [1]. It has been proposed that elevated gene expression in a particular environment likely contributes to fitness of that organism within that environment [2]. Consistent with this suggestion, several studies have identified niche-specific gene activation and subsequently demonstrated that mutations in some of those genes reduced fitness in the environment in question [3]–[6]. Using *in vivo* expression technology (IVET) to directly examine gene expression of *P. fluorescens* Pf0-1 in soil, we identified 22 genes which were up-regulated during growth in soil. Mutations were subsequently introduced into three of these genes, and in each case the mutant showed a defect in soil colonization. Interestingly, ten soil-induced antisense genes were discovered, none of which was predicted by computational annotation of the Pf0-1 genome sequence [1],[7]. These have previously been termed ‘cryptic’ genes as they had escaped detection by gene hunting algorithms [7]. Herein, these are termed ‘hidden’ or ‘non-predicted’ genes.

Although antisense transcription has been reported in eukaryotic systems [8]–[11], antisense genes in prokaryote genomes have received limited attention, usually as antisense RNA regulators [12],[13]. Previous IVET experiments have suggested the existence of additional sense/antisense transcriptional pairs in bacteria [e.g. 3,6], but these suggestions have not been further investigated.

We chose for further study six loci at which non-predicted antisense genes were reported opposite predicted genes. Here we report the confirmation of sense/antisense transcription at each of these six loci. We demonstrate that both the hitherto unknown gene *iiv14*, and the putative membrane protein gene found opposite, specify proteins. Further, we show that the gene *iiv14* is important for colonization of soil, and therefore name the *iiv14* gene *cosA.*

Our analysis of sense/antisense transcripts in *P. fluorescens* dramatically increases the number of experimentally verified sense/antisense pairs in bacteria. Our data suggest that bacterial genomes are more densely coded than currently known, and that key traits pertaining to microbial ecology can be specified by hidden genes found antisense to those predicted during genome annotation.

## Results

### Predicted Genes and Non-Predicted Antisense Genes in *P. fluorescens* Pf0-1 Are Transcribed

**Figure 1:**
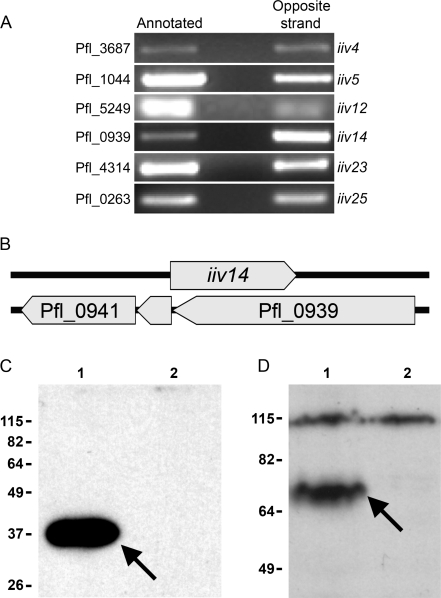
Transcription, organization, and translation of annotated and non-predicted antisense genes. A. RT-PCR, using gene-specific primers, of six pairs of annotated and antisense genes. At each locus, transcription from both DNA strands was detected. In all cases, negative controls which excluded reverse transcriptase from the reactions showed that contaminating DNA was not present. Pfl numbers refer to predicted Pf0-1 genes, (GenBank). The *iiv* numbers are as described [1]. B. Organization of the *iiv14* locus. Solid arrows indicate predicted open reading frames for annotated (Pfl ORFS) and cryptic (*iiv14*) genes. C. Western blot of purified iiv14-His protein. Lane 1: His-tagged iiv14 protein (arrow). Lane 2: control extract from Pf0-1(pME6000), showing no non-specific detection of untagged proteins. D. Western blot of His-tagged Pfl_0939. Crude extracts were prepared from Pf0-1 with and without Pfl_0939-His. Lane 1: His-tagged Pfl_0939 protein (arrow). Lane 2: extract from Pf0-1(pME6000) showing no non-specific detection of proteins similar in size to Pfl_0939. There is a non-specific background band at 115 kDa in both. Approximate sizes of molecular weight standards (Invitrogen BenchMark Pre-stained Protein Ladder) used in the SDS-PAGE are shown in panels C and D.

We previously reported the discovery of ten DNA sequences expressed during growth of *P. fluorescens* Pf0-1 in soil, that were antisense to predicted genes in the genome of Pf0-1 [1]. These ten antisense sequences are not physically linked to each other, and have no similarity to known protein-coding genes [1]. We carried out RT-PCR experiments at six loci using gene specific primers to generate the cDNA, and thus distinguish transcripts produced from the two DNA strands. In laboratory cultures we detected a basal level of expression from both the predicted coding and antisense sequences (Figure 1A), lower than that required for observable *dapB* reporter gene activity in the initial IVET screen and for survival on minimal medium [1]. In a control experiment at the *rpoS* locus, transcription was only detectable from the *rpoS* gene, not the opposite DNA strand (Figure S1). In all RT-PCR experiments, controls in which reverse transcriptase was omitted produced no products. The direct demonstration of transcription of the non-predicted antisense genes confirms their existence, and transcription of the predicted genes on the opposite strand indicates these are not simply mis-annotated.

### Transcriptional Mapping of the Non-Predicted Gene *iiv14*

The gene *iiv14* was chosen for further investigation. The transcribed region of *iiv14* identified by RT-PCR is within a potential open reading frame complementary to bases 1092441–1093457 in the Pf0-1 genome sequence, the conceptual translation product of which has no significant matches in GenBank (GenBank accession number CP000094). If the *iiv14* transcript spanned the ORF, it would suggest that *iiv14* could be translated. The transcription start site was mapped by 5′ RACE to 160 bp upstream of the candidate ORF. RT-PCR experiments with gene-specific primers were used to determine that the 3′ end of the transcribed sequence was at least 210 bp downstream of the presumed stop codon. In addition, the TransTermHP website (http://transterm.cbcb.umd.edu/tt/Pseudomonas_fluorescens_PfO-1.tt) showed a predicted terminator spanning bases 1092229–1092199 of the Pf0-1 genome (starting 193bp downstream of the presumed stop codon), with a confidence score of 41. Thus, the *iiv14* transcript is at least 1388 nucleotides long, and the candidate ORF is within the *iiv14* transcript (Figure S2).

### Both *iiv14* and the Predicted Gene Opposite Are Translated

To provide direct evidence for translation of the candidate *iiv14* ORF, and the predicted opposite gene (Pfl_0939) (Figure 1B) we added codons for six histidines to the 3′ end of both putative genes, cloned each in plasmid pME6000 (about 15 copies per cell), and transferred these to Pf0-1. Both constructs included the ORF plus upstream sequence likely to contain the native promoter (747 bp for *iiv14* and 148 bp for Pfl_0939). Inclusion of the native promoter and expression in Pf0-1 ensured that observed proteins were not artifacts resulting from expression in a heterologous host or under control of a non-native promoter. Western blot analysis using an antibody directed to polyHis demonstrated that both the *iiv14* ORF and Pfl_0939 specify proteins, of approximately the expected size (Figure 1C and D). The *iiv14* protein was 37 kDa, consistent with the predicted molecular weight (MW) of 38.788 kDa, while the MW of the Pfl_0939 protein was about 70 kDa, slightly less than the expected 80.745 kDa, but not unusual for membrane proteins. Consistent with the fact that *iiv14* is upregulated in soil and only expressed at a basal level in laboratory culture, the His-tagged *iiv14* protein had to be purified from 1L of culture and concentrated prior to western blot analysis, and films were exposed for 14–16 hours to obtain a clear signal.

### The *iiv14*/Pfl_0939 Locus Is Required for Efficient Soil Colonization

**Figure 2:**
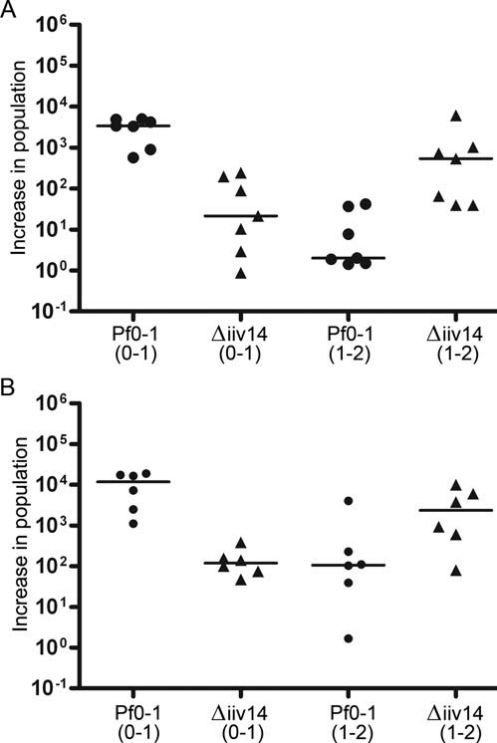
Soil colonization experiments. A. Growth of Pf0-1 wild-type and *iiv14* mutant strains (Δiiv14) in sterile soil, inoculated from laboratory culture. Data points represent fold increases from individual experiments, horizontal lines represent median values. 0–1, population increase between inoculation and day 1. The population increase of mutant Δ*iiv14* is significantly lower than that of Pf0-1 (p<0.001); 1–2, the population increase over the second day. The mutant Δ*iiv14* recovers from the colonization defect, and increases significantly more than Pf0-1 (p<0.005), which had already neared its maximum. B. Growth of Pf0-1 and the *iiv14* mutant in sterile soil, after prior growth in soil. Both strains were grown in separate soil samples for seven days. Populated soil was then mixed with fresh sterile soil to achieve dilution in the range of 1∶1000–1∶10000, and water was added to achieve approximately 50% water holding capacity. Populations in the fresh soil were monitored as described above. 0–1, population increase between inoculation and day 1. Over this period, Pf0-1 colonized the soil significantly better than the mutant Δ*iiv14* (p<0.005); 1–2, the population increase over the second day. The increases are not significantly different. Pf0-1 is represented by circles, while the *iiv14* mutant is indicated by triangles.

The proposed translation product of *iiv14* has no significant matches in BlastP searches of GenBank, providing no functional clues. We therefore sought to examine its importance in a sterile soil growth assay, where its expression is elevated. The *iiv14* ORF was deleted by SOE PCR [14] and allele exchange as described [1]. Because *iiv14* and Pfl_0939 overlap, the deletion results in a double mutant. Further, deletion of the *iiv14* ORF also removes the first 44 bases of Pfl_0940, probably rendering it non-functional. Growth of this deletion mutant in soil was monitored by periodic sampling of colony forming units. The *iiv14*/0939/0940 mutant was unable to colonize soil as rapidly as Pf0-1 (p<0.001) (Figure 2A, columns 1 and 2). However, between the first and second days when the wild-type had already approached maximum colonization density, the population of the Pf0-1Δ*iiv14* strain increased 1000 fold (Figure 2A, column 4), such that the cell numbers of both strains were approximately equal after two days of growth. Thus, the *iiv14*/0939/0940 deletion affected the early part of soil colonization, rather than soil survival *per se*. In laboratory medium (PMM), both Pf0-1 and the mutant showed approximately equal doubling times (65 min).

For the soil growth experiments, cells grown in laboratory culture were used as the inoculum. Thus, the slow initial growth of Pf0-1Δ*iiv14* could potentially be explained by a defect in adapting from laboratory media to soil. We therefore transferred wild-type and *iiv14*/0939/0940 mutant bacteria which had grown in soil for seven days into fresh soil, by diluting the previously colonized soil with fresh sterile soil. After one day in fresh soil, the increase of the mutant population was significantly (at least 100-fold) lower than that of Pf0-1 (p<0.005), demonstrating that the deletion of the *iiv14* locus results in a soil colonization defect, not a defect in adapting to growth in soil after growth in laboratory culture medium (Figure 2B). Over the period between 24 and 48 hours, the *iiv14-*locus mutant population increased more than the Pf0-1 population, as the latter had already approached its maximum density in the first 24 hours.

### Complementation Experiments Demonstrate *iiv14* Is Important in Soil Colonization

To be certain that the deletion of the *iiv14* locus caused the reduced soil colonization, we used allele exchange to replace the deleted region with wildtype sequence. To achieve this, region 1092301–1093786 in the Pf0-1 genome, which spans *iiv14*, was cloned in pSR47s. The resulting plasmid was used in allele exchange as described [1]. Recombinants possessing wildtype sequence were confirmed by PCR. In soil colonization experiments, the replacement strain was indistinguishable from Pf0-1, confirming that the deletion was completely responsible for the colonization defect (data not shown).

**Figure 3:**
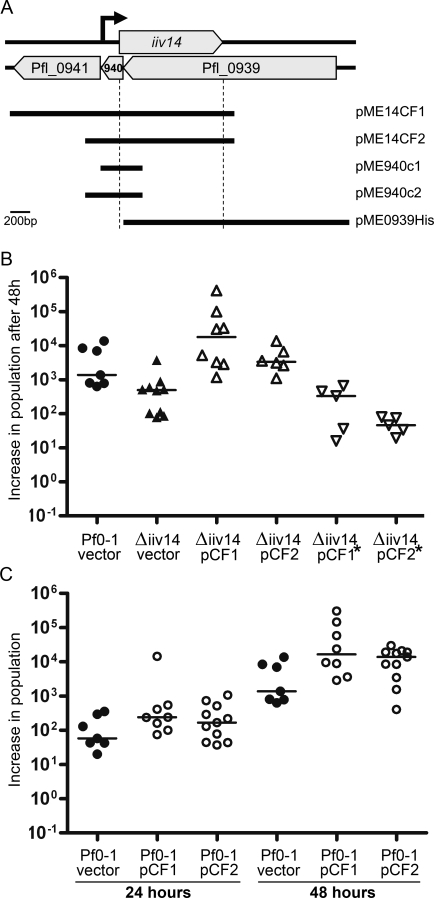
Restoring and enhancing soil colonization with multicopy clones of *iiv14*. A. Organization of *iiv14*, Pfl_0939, Pfl_0940, and Pfl_0941 in the Pf0-1 genome. Horizontal lines below indicate the cloned regions in each complementation construct. Vertical dotted lines show the boundaries of the *iiv14* ORF. The arrow indicates the location of the *iiv14* transcription start site. From this it can be seen that the only clones possessing the full length *iiv14* sequence are pME14CF1 and pME14CF2. B. Fold increase in population of the *iiv14* mutant (Δiiv14) bearing complementing plasmids pME14CF1 and pME14CF2, compared to mutant and wild-type strains harboring vector pME6000, and compared to the *iiv14* mutant harboring complementation plasmids with a stop codon replacing codon 17 of the gene, after two days growth in soil. The mutant Δ*iiv14* carrying complementing plasmids colonize significantly better than the mutant carrying the plasmid vector alone (pCF1, p<0.0005; pCF2, p<0.005). The mutant harboring plasmids carrying the nonsense codon (indicated by *) did not colonize significantly better than the mutant carrying the vector alone, and colonized significantly less than Pf0-1 harboring the vector (p<0.01). C. Effect of multiple copies of both *iiv14* gene clones in Pf0-1 on soil colonization, relative to Pf0-1(pME6000). Population increases over 24 and 48 hours, are shown. The greater colonization shown by Pf0-1 carrying pCF1 or pCF2 was significant over the 48 hour colonization period (columns 4–6; p<0.05). In panels B and C, data points represent fold increases from individual experiments, horizontal lines represent median values. Pf0-1 and the *iiv14* mutant are represented by circles and triangles, respectively. Inverted triangles represent strains in which the plasmid carries the stop codon at codon 17 of *iiv14*. Filled symbols show strains carrying pME6000 (vector), open symbols indicate carriage of complementing plasmids: pCF1 = pME14CF1; pCF2 = pME14CF2; stars show plasmids carrying stop codons at codon 17.

Because the two genes *iiv14* and Pfl_0939 overlap each other, deletion of one results in loss of the other. To test whether loss of *iiv14* was sufficient to explain the soil colonization defect, we cloned the *iiv14* ORF and upstream region into plasmid pME6000. Two versions of the complementation clone were constructed, one consisting of 1155bp upstream of the *iiv14* ORF (called pME14CF1), and the other containing only 329bp of upstream sequence, including 169bp upstream of the *iiv14* transcriptional start site (called pME14CF2) (Figure 3A). Neither of these clones includes the full-length Pfl_0939 coding sequence opposite *iiv14*. Thus, phenotypes attributed to the presence of the complementation clones are related to restoration of *iiv14*, not the opposite gene. *P. fluorescens* strains possessing the vector pME6000 grew slower than plasmid-free strains in soil. Therefore, complementation experiments were followed and analyzed on day two, rather than after one day in soil. The findings were compared to controls of Pf0-1 and Pf0-1Δ*iiv14* carrying the plasmid vector. As seen with plasmid-free strains (Figure 2), the Pf0-1(pME6000) population increased significantly more (about 10-fold; p<0.001) after one day than did the Pf0-1Δ*iiv14*(pME6000) population (not shown). After two days, the Pf0-1(pME6000) population had increased more than the *iiv14* mutant carrying pME6000 (p<0.05) (Figure 3B, column 1 and 2), verifying that the phenotypes associated with plasmid-free strains were true for plasmid-bearing strains. Both pME14CF1 and pME14CF2 complemented the defect in Pf0-1Δ*iiv14* (Figure 3B; compare column 2 with columns 3 and 4). Relative to Pf0-1Δ*iiv14* harboring the vector alone, the population of complemented strains increased significantly after two days in soil (p<0.005). To further test whether *iiv14* or Pfl_0939 was important in soil colonization, the plasmid constructed for expression of His-tagged Pfl_0939 protein (pME0939His) was utilized in complementation experiments. This plasmid lacks the coding sequence for the first 12 amino acids of *iiv14*, so only Pfl_0939 protein is made. Unlike pME14CF1 and pME14CF2, the pME0939His failed to restore soil colonization, demonstrating that the defect is not due to the loss of Pfl_0939 (data not shown).

As described above, deletion of *iiv14* also removed 44 base pairs of the Pfl_0940 coding sequence. Plasmids pME14CF1 and pME14CF2 both contain the coding sequence for Pfl_0940 in addition to *iiv14* (Figure 3A). To distinguish between the deletion of *iiv14* and disruption of Pfl_0940 as the cause for the soil colonization defect, we constructed two additional complementation clones in pME6000, called pME940c1 and pME940c2, both of which include the full length of Pfl_0940, but not *iiv14* or Pfl_0939. (Figure 3A). Neither of these clones was capable of restoring the soil growth phenotype of the *iiv14*/0939/0940 mutant (data not shown). Taken together, the complementation experiments using plasmid-based constructs demonstrate that of the three genes at the *iiv14* locus, it is the non-predicted *iiv14* that is important in colonization of soil, and was designated *cosA*.

### A Nonsense Codon in *cosA* Abolishes Complementation

Having demonstrated that *cosA* is important for soil colonization, and that the gene specifies a protein, we created a nonsense mutation in the gene to determine whether it was the *cosA-*specified protein or some other feature of the sequence that was important for soil colonization. Codon 17 of the *cosA* gene was changed from AAG to TAG, after which the mutated DNA region was cloned to create plasmids that were identical to pME14CF1 and pME14CF2, apart from the nonsense mutation. The AAG to TAG change in *cosA* results in a silent change from leucine codon CTT to leucine codon CTA in the Pfl_0939 sequence on the opposite strand. In the sterile soil colonization assay, the mutation-containing plasmids were unable to complement the colonization defect, relative to Pf0-1(pME6000) (p<0.01) (Figure 3B, columns 5 and 6).

### Multicopy *cosA* Clones Improve Soil Colonization

Deletion and complementation experiments (above) demonstrated that *cosA* specifies a soil colonization factor. Multiple copies of *cosA* (on the complementing plasmids) accelerated soil colonization by wild-type Pf0-1. After one day in sterile soil, the population of Pf0-1 carrying pME14CF1 increased significantly more than that of Pf0-1 carrying the vector alone (p<0.05) (Figure 3C, columns 1 and 2). Over the first two days in soil, the median increase of the Pf0-1(pME14CF1) population was more than 15 times that of Pf0-1(pME6000), while the median increase for Pf0-1(pME'14CF2) was about 10 times that of the control (p<0.05 for both) (Figure 3C, columns 4–6).

### *cosA* Mutants Have a Competitive Defect

**Figure 4:**
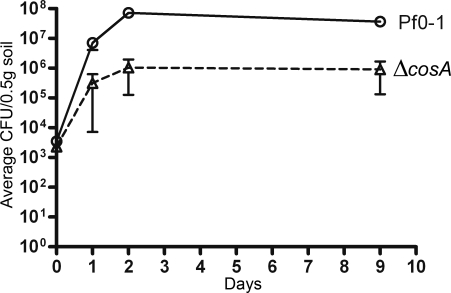
Soil competition experiments. Pf0-1 and the *cosA* mutant strains marked with Sm^r^ (Pf0-1) or Km^r^ (Δ*cosA*) carried on miniTn*7* were each diluted to contain approximately 10^4^ cfu/mL. Soil was inoculated with 1mL of a 50∶50 mix of each. Populations were monitored daily by cfu counting. Data shown are the average cfu/0.5 g soil, from four independent experiments. Error bars represent the standard error of the mean.

When Pf0-1Δ*cosA*km^r^ was introduced into sterile soil in competition with wild-type Pf0-1Sm^r^, the mutant was not competitive during the first day, and was unable to increase its relative population over subsequent days (Figure 4). This result is in contrast to competitions between differently marked wild-type strains, where each makes up 50% of the soil population after co-inoculation with equal numbers (not shown). The proportion of Pf0-1Δ*cosA* in the population did not decline over time, confirming that the fitness defect of the mutant is confined to the early colonization period.

## Discussion

Our results demonstrate that there are at least six loci in *P. fluorescens* Pf0-1 at which both annotated (‘sense’) and ‘antisense’ DNA sequences are transcribed. Further investigation of a chosen example provided conclusive evidence for overlapping protein-coding genes specified opposite each other by the same stretch of DNA. Importantly, a role for this locus in soil colonization was identified, and of the two overlapping genes present, it was the novel non-predicted gene *cosA* that was required for efficient soil colonization.

These findings suggest that current genome annotations provide an incomplete view of the genetic potential of a given organism, a proposal that is not without precedent in prokaryotic biology. Genes specifying the small ncRNAs affect processes including transcriptional regulation, mRNA stability, and chromosome replication [15]. These are still difficult to predict *ab initio* in organisms for which there is little information on promoter consensus sequences, although new computational approaches have recently become available [16]. In the prokaryotic horizontally mobile elements, antisense RNA has long been known to control copy number in plasmids, and play a role in bacteriophage development [reviewed in 17]. For prokaryote chromosomal genes, both *trans*- and *cis*-encoded antisense RNA regulators are known: e.g. control of *glnA* in *Clostridium acetobutylicum* [18], *ompF* in *E. coli* [19], and the photosynthesis gene *isiA* in *Synechocystis* sp. PCC 6803 [13].

In eukaryotes, the concept that genomes include numerous sense/antisense gene pairs is becoming increasingly obvious with genome-wide transcriptional studies in yeast [8] and *Arabidopsis* [10]. Antisense transcripts have been implicated in eye development [20] and control of entry into meiosis in yeast [21]. However, discussion of antisense transcription is limited to possible regulatory roles for antisense RNA [e.g. 8], without consideration of the possibility that they may specify proteins. Genome annotations do not routinely predict the existence of two protein-coding genes on opposite DNA strands, and in fact normally deliberately eliminate predicted overlaps. Moreover, small protein-coding genes can be missed by predictive algorithms. For example, the *blr* gene in *E. coli* specifies a 41 residue protein, and was discovered in a sequence believed to be intergenic [22].

The fact that antisense genes have been implicated in important biological functions indicates that more attention should be given to this emerging class of genes. Because they are difficult to predict with existing algorithms, experimental techniques should be adapted to allow their inclusion in genome-wide surveys. Tiling array technology is not biased toward predicted genes, and thus can be used to reveal the existence of non-predicted transcripts, such as those found antisense to known genes. Proteomic studies using accurate mass tags have the capacity to identify any and all proteins produced by a given organism. If data from such experiments are analyzed in an unbiased way, proteins produced from non-predicted genes will be identified. Finally, genetic techniques such as the IVET approach that led to the discovery of the *iiv* sequences described here [1] are well-suited to the discovery of novel genes. We have argued previously [7] that the frequency with which antisense genes are detected by promoter trapping strongly suggests that they represent real genes. The added advantage of IVET-like experiments is that they provide information regarding up-regulation in a particular environment, which yields clues as to function.

The exact role for *cosA* in colonization of soil is currently unknown. The *cosA* deletion mutant has no growth defect in laboratory culture, yet is impaired in soil colonization. Pf0-1 strains possessing the complementing *cosA* containing plasmids are more rapid colonizers of soil than the control strains (Figure 3C), but do not grow faster than control strains in laboratory media. In fact, Pf0-1 carrying the plasmid pME14CF1 forms colonies on agar surfaces considerably less quickly than control strains.

Two proteins, SetA (20 kDa) and SetB (7 kDa), [23] are thought to be encoded opposite *pic*, which specifies a serine protease [24] on a pathogenicity island in *Shigella flexneri* serotype 2 strains [25],[26] and in enteroaggregative *E. coli* [24],[27]. Thus, this example of probable antisense protein-coding genes evolved in the context of a horizontally mobile element.

We have demonstrated that at one sense/antisense chromosomal locus (*cosA*), both the predicted ‘sense’ gene (Pfl_0939) and the unpredicted ‘antisense’ gene *cosA* specify proteins, and it is the non-predicted gene identified initially from gene-expression in soil studies which is important for colonization of soil in a model laboratory system. Thus, antisense genes may be more functionally diverse than simply making regulatory or antisense RNAs. The *cosA/*Pfl_0939 pair is the first demonstration of overlapping antisense protein-coding genes in a prokaryote genome.

## Materials and Methods

### Bacterial Strains and Plasmids

*Pseudomonas fluorescens* Pf0-1 was used as wild-type [28]. The genome sequence of *Pseudomonas fluorescens* Pf0-1 is available under GenBank accession number CP000094. Pf0-1Δ*iiv14/cosA* was made by deleting bases 1092441 to 1093457 in the Pf0-1 genome by SOE-PCR [14] and allele exchange using plasmid pSR47s [29], as described [1]. *E. coli* DH5α (F- φ80*lac*ZΔM15 Δ(*lac*ZYA-*arg*F) U169 *rec*A1 *end*A1 *hsd*R17 (r_k_−, m_k_+) *pho*A *sup*E44 λ- *thi*-1 *gyr*A96 *rel*A1) (Invitrogen, Carlsbad, CA) was used to propagate plasmids. *E. coli* S17-1 (*recA pro hsdR* RP4-2-Tc::Mu-Km::Tn*7* λ-*pir*) [30] served as the donor strain in conjugations. *P. fluorescens* strains were grown in PMM [31] at 30°C, and *E. coli* strains were grown in LB at 37°C. Plasmid pME6000 [32] was used for complementation studies and to express His-tagged proteins. Streptomycin resistant miniTn*7* was constructed by inserting a Sm^r^ cassette from pHRP315 [33] into pUCT-mTn7T [34], while kanamycin resistant MiniTn*7* is carried on pHRB2 [35]. MiniTn*7* constructs were used to introduce Km^r^ and Sm^r^ markers into *P. fluorescens* strains for competition experiments, as described [36]. Complementation plasmids pME14CF1 and pME14CF2 contain regions 1092301–1094612 and 1092301–1093786 of the Pf0-1 genome, respectively. Plasmids pME940c1 and pME940c2 contain regions 1093208–1093627 and 1093208–1093786, respectively. Plasmid pME14His contains bases 1092438–1094204 of the Pf0-1 genome, and has codons for 6-His introduced immediately upstream of the *cosA* stop codon. Plasmid pME0939His contains bases 1091084–1093421 of the Pf0-1 genome and has codons for 6-His introduced immediately upstream of the Pfl_0939 stop codon. Pf0-1 sequences in each clone were amplified by PCR. In both cases, the six histidine codons were added by inclusion in the 3′ primer used to amplify the desired sequences.

### DNA Manipulation and Sequencing

Recombinant DNA techniques were carried out as described [37]. Restriction and DNA modifying enzymes were purchased from Invitrogen Inc and New England Biolabs (Beverly, MA). Plasmid DNA was purified using the Qiaprep spin miniprep kit (Qiagen, Valencia, CA). Genomic DNA was prepared using Promega's Wizard Genomic DNA purification kit (Madison, WI). DNA was recovered from agarose gel slices using the Qiaex II gel extraction kit (Qiagen). PCR was carried out with Invitrogen Platinum *Taq* DNA polymerase High Fidelity. PCR products were cloned with pGEM-T Easy (Promega). Oligonucleotides were purchased from IDT (Coralville, IA) and DNA sequences were determined at the Tufts University Core Facility (Boston, MA).

### Introduction of Nonsense Codon into *cosA*

The complementing regions from pME14CF1 and pME14CF2 were cloned into plasmid pHRB2, which is smaller than the pME6000 vector. Codon 17 was changed from AAG to TAG. This also causes a silent change from leucine codon CTT to leucine codon CTA in Pfl_0939. The nonsense mutation was introduced using the “round the horn” protocol as described (http://openwetware.org/wiki/'Round-the-horn_site-directed_mutagenesis). The DNA polymerase used was KOD Hot Start DNA polymerase (Novagen). Primers used were 14mutF (tgggcTagtccttcgggcttg), which contains the base change to create the nonsense mutation (upper case), and 14wtR2 (tgcctcgtgaaatcgccttcc). The nonsense mutation was verified in resulting plasmids by DNA sequencing. Two mutants of each complementing region were then each recloned into pME6000, and the DNA sequence was again verified. Each of the four resulting plasmids (two for each of CF1 and CF2) were transferred to Pf0-1Δ*cosA*, and then assessed for complementing ability in the soil assay as described below.

### Soil Growth and Survival

Soil growth and survival assays were carried out as described previously [1] using gamma-irradiated, sandy loam soil, of known composition [38]. Briefly, cultures were grown for 16 hours in minimal medium, and diluted to contain approximately 10^4^ cfu/mL. One mL of diluted culture was mixed with 5 g of soil, achieving approximately 50% water-holding capacity. Cultures for competition experiments in soil were adjusted to equal A_600_ values prior to dilution and then 500 µL of each competitor were used to inoculate soil as above. Population increase was monitored over time by extraction of cells and cfu determination by colony counting on selective media.

### RNA Isolation and RT-PCR

*P. fluorescens* Pf0-1 RNA was isolated using an RNeasy Mini Kit, including the on-column DNaseI treatment (Qiagen). The RNA was then treated with RQ1 DNaseI (Promega) for 1h at 37°C, and re-purified using a Qiagen RNeasy column. For RT-PCR experiments, cDNA was synthesized from 500 ng of total RNA using Superscript III (Invitrogen) and a gene specific primer, at 52°C for 1 h. The cDNA was amplified by PCR using the gene specific primer, and an appropriate partner primer. All RT-PCR experiments were carried out with a negative control consisting of a reverse transcriptase-free reaction.

### 5′ RACE

5′ RACE was carried out using Invitrogen 5′ RACE system as recommended. Gene specific primers were 14RT-R (5′-ggcctgctgatctttttcag), 14GSP2 (5′-tgttcctgcaaccgaattcg), and 14GSP3 (5′-gggtgaaaagctacctgcac). Products were cloned in pGEM-T Easy, and sequenced using T7 and SP6 primers.

### Protein Purification and Western Blotting

Proteins specified by *cosA* and Pfl_0939 were modified by addition of six histidine codons immediately before the stop codon, by PCR. In all cases, cultures were grown to A_600_ = 0.4, in PMM. His-tagged CosA protein was extracted and purified from *P. fluorescens* Pf0-1 carrying plasmid pME14His, using denaturing conditions, as described for *E. coli* in the QIAexpressionist handbook (Qiagen). Amicon Ultra-4 (10k) (Millipore, Billerica, MA) centrifugal filters were used to concentrate proteins. His-tagged Pfl_0939 was extracted from Pf0-1 carrying plasmid pME0939His, by sonication of cells, and solubilization in SDS buffer [37], resulting in a crude extract which was used in the western blots. Controls for each extraction (Pf0-1 carrying vector alone) were processed in the same way as each experimental preparation.

For western blot analysis, proteins were separated by SDS-PAGE (12.5% acrylamide for CosA-His protein, and 10% for Pfl_0939-His) and transferred to PolyScreen PVDF membrane (Perkin Elmer, Waltham, MA) using a Biorad Mini Trans-blot Cell (70 V, 1 h). Membranes were processed for immunodetection as described (QIAexpress Detection and Assay Handbook). Mouse monoclonal anti-polyHistidine antibody (Sigma, St. Louis, MO) was diluted 1∶10000 in fresh TTBS (17.2 mM NaCl, 5.1 mM KCl, 24.8 mM tris base, 22 mM HCl (to pH 7.4), 0.1% Tween 20)+3% BSA. HRP-linked antimouse IgG antibody (Cell Signaling Technology, Danvers, MA) secondary antibody was diluted 1∶10000 in TTBS plus 10% non-fat milk powder. Detection of antibodies was carried out using Western Lightning Western Blot Chemiluminescence Reagent *Plus* (Perkin Elmer). Luminescence was detected with Kodak BioMax MR film after 18 h of exposure. Protein molecular weights were estimated by comparison to the BenchMark Pre-Stained Protein Ladder (Invitrogen).

### Statistical Analysis

Data from soil experiments were analyzed by the Mann Whitney test using GraphPad Prism 4 software.

## Supporting Information

Figure S1 RT-PCR at the *rpoS* locus using strand-specific primers. Using the same method as was used for the sense/antisense pairs, we carried out RT-PCR at the *rpoS* locus as a negative control. In contrast to the sense/antisense pairs shown in Figure 1, transcription could only be detected from the annotated (*rpoS*) gene, and not from the opposite DNA strand, indicating that this approach successfully discriminates between loci in which one or both strands are transcribed.
(4.04 MB TIF)

Figure S2 Mapping the *iiv14/cosA* transcript. A. Location of the *iiv14/cosA* transcription start site (+1), identified by 5′RACE, relative to a predicted ORF in the Pf0-1 genome (bases 1093457–1092441) (shaded arrow). Also shown are the approximate locations of the primers (F and R) used to show transcription by RT-PCR. The vertical bar at the 3′ end indicates an arbitrary downstream boundary for searching for the transcription terminator. B. Region from base 1180 to 1503, relative to the +1 site. Gene specific RT-PCR primers 1–4, used to map the 3′ end of *iiv14/cosA*, are shown. C. RT-PCR to map the 3′ end of the *iiv14/cosA* transcript. RT was carried out with gene specific primers 1–4, followed by PCR with the gene specific primer and primer ‘F’. RT-PCR products are in lanes marked ‘+’, reverse transcriptase-free negative controls are in ‘−’ lanes, while the ‘C’ lanes show positive control PCR using genomic DNA as the template. D. Region 1374–1434, showing the DNA sequence of a putative transcriptional terminator identified by TransTerm. The ‘t’ underlined in the left stem (arrowed) does not match, and is predicted to bulge out of the stem.
(3.51 MB TIF)
