# Supplementary figures and images for: Overlapping Protein-Encoding Genes in Pseudomonas fluorescens Pf0-1
Source: PLoS Genet. 2008 Jun 13;4(6):e1000094. doi: 10.1371/journal.pgen.1000094 (PMC2396522; doi:10.1371/journal.pgen.1000094)

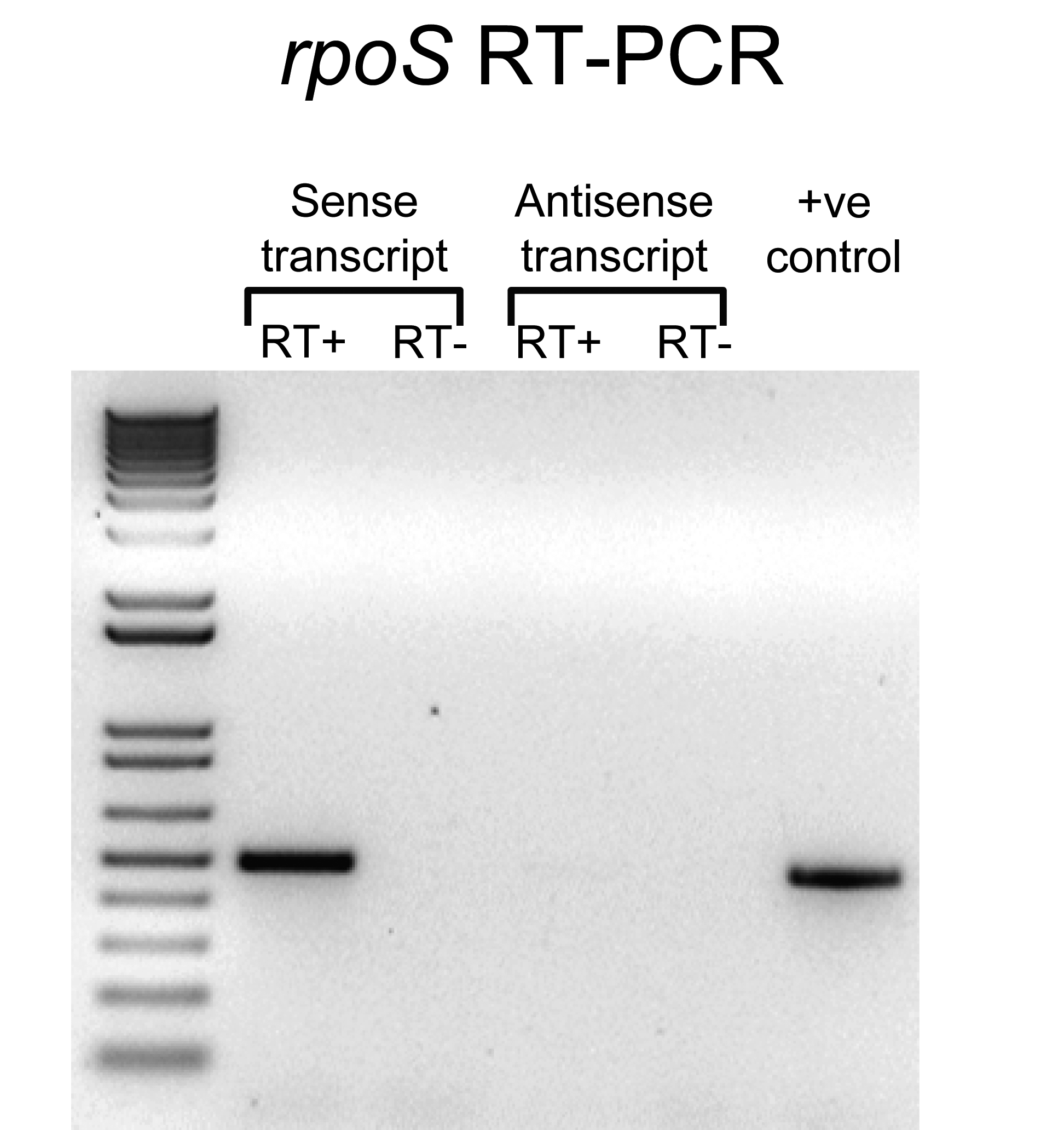

Supplement: Figure S1 — RT-PCR at the rpoS locus using strand-specific primers. Using the same method as was used for the sense/antisense pairs, we carried out RT-PCR at the rpoS locus as a negative control. In contrast to the sense/antisense pairs shown in Figure 1, transcription could only be detected from the annotated (rpoS) gene, and not from the opposite DNA strand, indicating that this approach successfully discriminates between loci in which one or both strands are transcribed. (4.04 MB TIF) [file pgen.1000094.s001.tif]

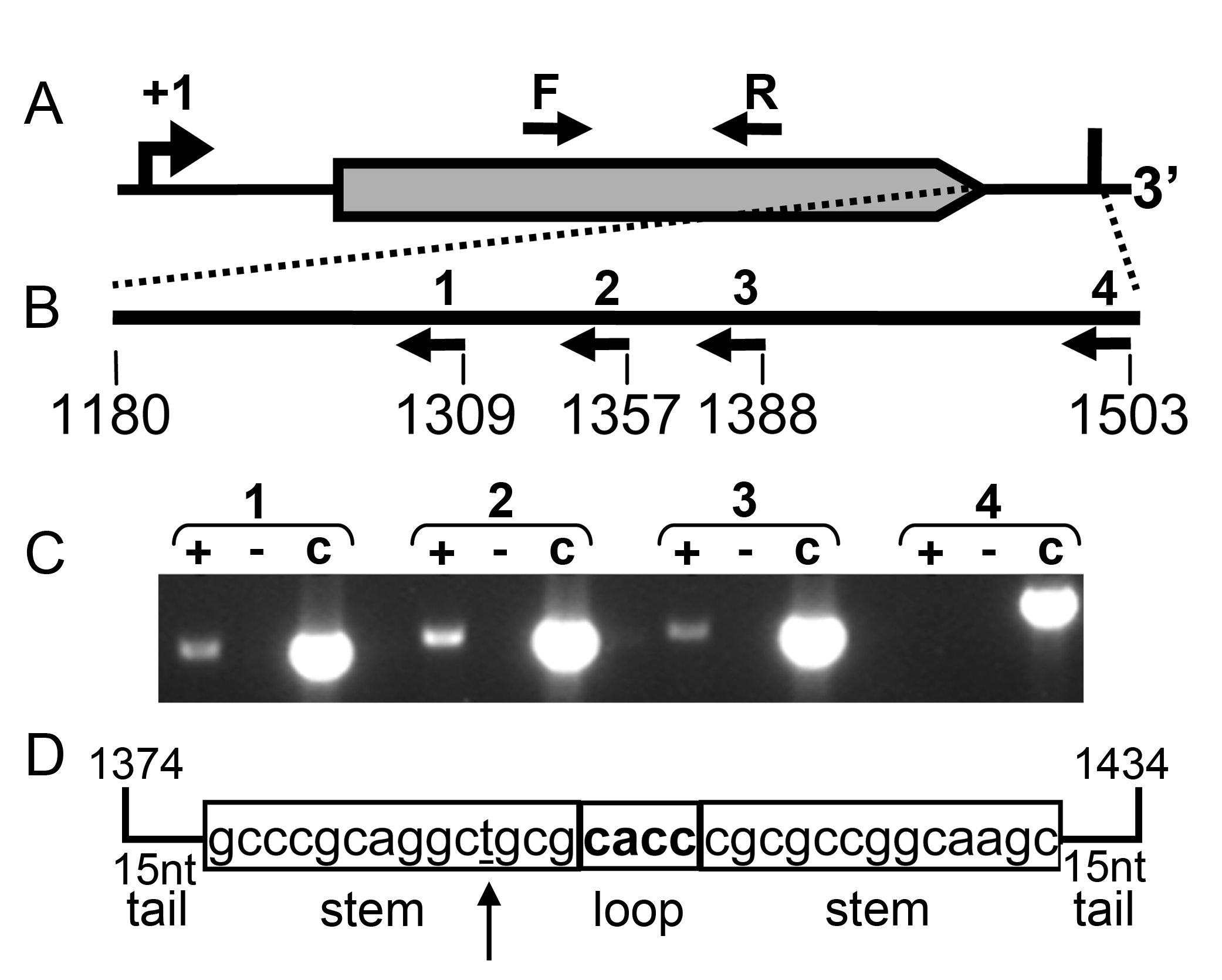

Supplement: Figure S2 — Mapping the iiv14/cosA transcript. A. Location of the iiv14/cosA transcription start site (+1), identified by 5′RACE, relative to a predicted ORF in the Pf0-1 genome (bases 1093457–1092441) (shaded arrow). Also shown are the approximate locations of the primers (F and R) used to show transcription by RT-PCR. The vertical bar at the 3′ end indicates an arbitrary downstream boundary for searching for the transcription terminator. B. Region from base 1180 to 1503, relative to the +1 site. Gene specific RT-PCR primers 1–4, used to map the 3′ end of iiv14/cosA, are shown. C. RT-PCR to map the 3′ end of the iiv14/cosA transcript. RT was carried out with gene specific primers 1–4, followed by PCR with the gene specific primer and primer ‘F’. RT-PCR products are in lanes marked ‘+’, reverse transcriptase-free negative controls are in ‘−’ lanes, while the ‘C’ lanes show positive control PCR using genomic DNA as the template. D. Region 1374–1434, showing the DNA sequence of a putative transcriptional terminator identified by TransTerm. The ‘t’ underlined in the left stem (arrowed) does not match, and is predicted to bulge out of the stem. (3.51 MB TIF) [file pgen.1000094.s002.tif]
